# Prenatal Phthalate Exposures and Anogenital Distance in Swedish Boys

**DOI:** 10.1289/ehp.1408163

**Published:** 2014-10-29

**Authors:** Carl-Gustaf Bornehag, Fredrik Carlstedt, Bo AG. Jönsson, Christian H. Lindh, Tina K. Jensen, Anna Bodin, Carin Jonsson, Staffan Janson, Shanna H. Swan

**Affiliations:** 1 Department of Health Sciences, Karlstad University, Karlstad, Sweden; 2 County Council of Värmland, Karlstad, Sweden; 3 Division of Occupational and Environmental Medicine, Lund University, Lund, Sweden; 4 Department of Environmental Medicine, University of Southern Denmark, Odense, Denmark; 5 Icahn School of Medicine at Mount Sinai, New York, New York, USA

## Abstract

Background: Phthalates are used as plasticizers in soft polyvinyl chloride (PVC) and in a large number of consumer products. Because of reported health risks, diisononyl phthalate (DiNP) has been introduced as a replacement for di(2-ethylhexyl) phthalate (DEHP) in soft PVC. This raises concerns because animal data suggest that DiNP may have antiandrogenic properties similar to those of DEHP. The anogenital distance (AGD)—the distance from the anus to the genitals—has been used to assess reproductive toxicity.

Objective: The objective of this study was to examine the associations between prenatal phthalate exposure and AGD in Swedish infants.

Methods: AGD was measured in 196 boys at 21 months of age, and first-trimester urine was analyzed for 10 phthalate metabolites of DEP (diethyl phthalate), DBP (dibutyl phthalate), DEHP, BBzP (benzylbutyl phthalate), as well as DiNP and creatinine. Data on covariates were collected by questionnaires.

Results: The most significant associations were found between the shorter of two AGD measures (anoscrotal distance; AGDas) and DiNP metabolites and strongest for oh-MMeOP [mono-(4-methyl-7-hydroxyloctyl) phthalate] and oxo-MMeOP [mono-(2-ethyl-5-oxohexyl) phthalate]. However, the AGDas reduction was small (4%) in relation to more than an interquartile range increase in DiNP exposure.

Conclusions: These findings call into question the safety of substituting DiNP for DEHP in soft PVC, particularly because a shorter male AGD has been shown to relate to male genital birth defects in children and impaired reproductive function in adult males and the fact that human levels of DiNP are increasing globally.

Citation: Bornehag CG, Carlstedt F, Jönsson BA, Lindh CH, Jensen TK, Bodin A, Jonsson C, Janson S, Swan SH. 2015. Prenatal phthalate exposures and anogenital distance in Swedish boys. Environ Health Perspect 123:101–107; http://dx.doi.org/10.1289/ehp.1408163

## Introduction

Diesters of 1,2-benzenedicarboxylic acid (phthalic acid), commonly referred to as phthalates, belong to a group of chemicals with endocrine-disrupting properties, meaning that they can interfere with our normal hormonal balance, potentially resulting in adverse human health effects. A recent state-of-the-science report from the World Health Organization (WHO) provides new evidence for several human health risks from exposure to phthalates and other endocrine disruptors (EDCs) including reproductive health and sexual development (Bergman et al. 2013).

Phthalates are used as plasticizers in soft polyvinyl chloride (PVC) and found in a large number of commonly used consumer products including food, building materials, plastics, cosmetics, cleaning products, packages, and toys (Bornehag et al. 2005; Dodson et al. 2012; Rudel et al. 2011). Often, the fraction of phthalates in the plastics is as high as ≥ 30%. Because they are not covalently bound to the product matrix, phthalates can leach into the surrounding environment. As a result they are routinely found in indoor air (Bergh et al. 2011; Rudel and Perovich 2009) and dust (Abb et al. 2009; Bornehag et al. 2004, 2005; Langer et al. 2010; Zhang et al. 2013), and food and water (Shi et al. 2012).

Phthalates are ubiquitous in the environment, and humans are exposed by multiple routes (oral, dermal, and inhalation), the pathway varying by phthalate. It has recently been shown, for example, that exposure to di(2-ethylhexyl) phthalate (DEHP) is primarily via food and for children also via mouthing of plastics, whereas exposure to diethyl phthalate (DEP) and benzylbutyl phthalate (BBzP) occurs primarily via dermal and inhalation pathways (Carlstedt et al. 2013; Koch et al. 2013; Wittassek et al. 2011). Robust biomonitoring data show phthalate metabolites in human fluids such as urine, both in children (Langer et al. 2014) and adults (Wittassek et al. 2011), blood (Frederiksen et al. 2010; Wan et al. 2013), and breast milk (Fromme et al. 2011). These compounds have also been found in amniotic fluid, suggesting that they cross the placental barrier and expose the fetus (Jensen et al. 2012).

One complication in studying phthalate exposure is that replacement of phthalates is continuously ongoing as risks are reported for older formulations. One example of this is the introduction of diisononyl phthalate (DiNP) and more recently diisononyl cyclohexane-1,2-dicarboxylate (DINCH) to replace DEHP in soft PVC [European Chemicals Agency (ECHA) 2013]. Recent biomonitoring data show that urinary levels of DEHP metabolites are decreasing while DiNP metabolites are increasing in European (Göen et al. 2011) and American populations (Silva et al. 2013).

*Phthalates and reproductive health*. The impact of endocrine-disrupting chemicals on male reproductive health has been a research focus for almost 20 years. During that time, multiple studies in laboratory animals (since 1995) (Sharpe et al. 1995) and in humans (since 2005) (Swan et al. 2005) have demonstrated the sensitivity of the developing male reproductive system to several phthalates. Phthalates may reduce the production of androgens by the testis and rodent studies have demonstrated that DEHP and dibutyl phthalate (DBP) disrupt androgen signaling when administered in the critical window for the development of the reproductive tract (MacLeod et al. 2010; van den Driesche et al. 2011). In humans, similar findings suggest that phthalates (e.g., DEHP and DBP) may be related to male reproductive and developmental abnormalities (ECHA 2013).

*Anogenital distance and phthalate exposure*. Anogenital distance (AGD)—the distance from the anus to the genitals—is a marker that has been used in animal studies to assess reproductive toxicity (U.S. Environmental Protection Agency 1996). AGD is a sexually dimorphic trait that develops *in utero* under androgen control and is 50–100% longer in males than females.

Numerous studies have shown that prenatal phthalate exposure (notably DEHP, DBP, BBzP) shortens male AGD in rodents (Foster 2005; van den Driesche et al. 2011). Only a few human studies have examined prenatal phthalate exposure and AGD. The first to examine this association in humans reported significant inverse relationships between male AGD and DEP, DBP, BBzP, and mono-isobutyl phthalate (MiBP) metabolites (Swan et al. 2005); and a later publication with a larger sample size and more powerful statistical methods found associations between male end points and metabolites of DEHP (Swan 2008). Recently a relationship between prenatal DEHP exposure and shorter AGD in male newborns was reported from Japan (Suzuki et al. 2012) and from Mexico (Bustamante-Montes et al. 2013). However, a study from Taiwan found a negative association between prenatal monobutyl phthalate (MBP) exposure and AGD in newborn girls but not in boys (Huang et al. 2009). To date no studies in humans have included metabolites of DiNP.

*Aim of the study*. Animal data suggest that DiNP may have antiandrogenic properties similar to those of DEHP (Boberg et al. 2011). This raises concerns because DiNP has been introduced to replace DEHP, and consequently DiNP exposure is rapidly increasing in populations globally. Although several studies have examined phthalates and AGD in humans, none has included DiNP. Therefore, in the present study we examined the relationship between first-trimester urinary metabolite concentrations of DEP, DBP, DEHP, and BBzP as well as DiNP in relation to anogenital distance in boys at 21 months of age.

## Methods

*Description of the SELMA study*. The Swedish Environmental Longitudinal, Mother and child, Asthma and allergy (SELMA) study is a prospective birth cohort study in Sweden that includes > 2,000 mother–child pairs followed from early pregnancy (Bornehag et al. 2012). The study aim was to investigate the impacts of early-life exposure to endocrine-disrupting chemicals and other exposures, for multiple outcomes of growth, development, and chronic diseases.

The SELMA cohort was established by recruiting women in the 10th week of pregnancy in the county of Värmland, Sweden, between September 2007 and March 2010. Of 8,394 reported pregnant women, 6,658 were invited to participate in the study. Among the invited women, 2,582 agreed to participate, corresponding to a participating rate of 39%. Of the 4,076 nonparticipants, 2,091 women were invited to complete a nonrespondent questionnaire for us to examine possible selection bias. We found a self-selection bias in the established cohort when compared with the nonparticipant group—participating families smoked less (14% vs. 19%), had more frequent asthma and allergy symptoms in the family (58% vs. 38%), had higher education (university level) among the mothers (51% vs. 36%), and more often lived in single-family houses (67% vs. 60%). However, there was no obvious reason that this selection bias would have an impact on identification of environmental risk factors for health effects. Detailed recruitment procedures, selection criteria, and data relating to selection bias have been reported and discussed elsewhere (Bornehag et al. 2012). Biological samples (blood and urine) have been collected from the 2,582 pregnant women and their children. Information related to lifestyles, socioeconomic status, living conditions, diet, and medical history has been collected using annual questionnaires (Bornehag et al. 2012).

*Measurements of AGD in baby boys*. Boys born between 1 September 2009 and 20 November 2010 (i.e., those who were < 18 months of age in the SELMA study) (*n* = 325) and their parents were invited to participate in the study; of these, 228 participated (70%). Of these we were able to measure AGD in 225 boys, and of these, 196 children are included in the present analyses. Mean age of the 196 boys was 20.8 months. AGD was measured by two nurses (one pediatric staff nurse and one midwife) from the County Council of Värmland who were trained by research staff from The Infant Development and the Environment Study (TIDES) (Barret et al. 2014). The nurses had no knowledge of the mother’s phthalate concentrations. All AGD measurements were made in the baby’s home or at a pediatric clinic.

We obtained two measurements of AGD using measurement methods that have been described elsewhere (Sathyanarayana et al. 2010). Briefly, the longer AGD measurement (AGDap) was measured from the center of the anus to the anterior base of the penis, and the shorter (AGDas) from the center of the anus to the posterior base of the scrotum. The infant was placed on his back on a flat surface with his hips relaxed outward and pulled back towards his shoulders by his mother or an assistant. The examiner stood in front of the infant and made three measurements of both long and short AGD with a dial caliper; the coefficient of variation (CV) was calculated for each AGD measure. Only one measurement was made for 12 boys regarding AGDas and for 24 boys regarding AGDap.

*Phthalate metabolites in first-trimester urine*. Of the 2,582 pregnant women participating in the SELMA study, a first morning urine sample was obtained from 2,356 women (91%) during weeks 9–11 of the pregnancy. The urine samples were stored frozen at –20°C. These samples were analyzed for the 10 phthalate metabolites listed in Table 1. Briefly, 0.2 mL of urine were added with 0.1 mL of ammonium acetate (pH 6.5) and 0.01 mL glucoronidase (*Escherichia coli*) and thereafter incubated at 37°C for 30 min. Then we added 0.05 mL of a 50:50 (vol:vol) water and acetonitrile solution of labeled (^3^H or ^13^C) internal standards of all analyzed compounds. A C18 column (2.1 mm i.d. × 50 mm; Genesis Lightn; Grace, Deerfield, IL, USA) was used before the injector to reduce the interference of contaminants during the mobile phase. The phthalate metabolites in the samples were separated on a C18 column (1.5 μm, 2.0 mm i.d. × 30 mm VisionHT; Grace). The mobile phases were water and acetonitrile with 0.08% formic acid. The samples were analyzed on a Shimadzu UFLC system (Shimadzu Corporation, Kyoto, Japan) coupled to a QTRAP5500 triple quadrupole linear ion trap mass spectrometer equipped with a TurboIon Spray source (AB Sciex, Foster City, CA, USA). The samples were analyzed in triplicate, and the mean of the two closest were reported. All samples were analyzed in a randomized order. For quality control of the analyses, chemical blanks and two different in-house prepared quality control samples were analyzed in all sample batches. The limit of detection (LOD) was defined as the concentration corresponding to a peak area ratio of three times the standard deviation of the chemical blanks, and is shown in Table 1. The laboratory at Lund University is a reference laboratory for analyses of urinary phthalate metabolites in a European biomonitoring project [Consortium to Perform Human Biomonitoring on a European Scale (COPHES); http://www.eu-hbm.info/cophes]. The creatinine concentrations were analyzed according to an enzymatic method described by Mazzachi et al. (2000). We calculated the sum of DEHP and DiNP metabolites by summing the metabolite concentrations on a molar basis.

**Table 1:** Analyzed phthalate metabolites in the urine of 196 pregnant women, their parent compounds including CAS (Chemical Abstracts Service) number, and limit of detection (LOD).

Parent compound		Metabolite			LOD (ng/mL)
Diester name (acronym)	CAS no.	Monoester name	Acronym	CAS no.	
Diethyl phthalate (DEP)	84-66-2	Mono-ethyl phthalate	MEP	2306-33-4	0.01
Dibutyl phthalate (DBP)	84-74-2	Mono-*n*-butyl phthalate	MnBP	131-70-4	0.10
Butylbenzyl phthalate (BBzP)	85-68-7	Mono-benzyl phthalate	MBzP	2528-16-7	0.04
Di(2-ethylhexyl) phthalate (DEHP)	117-81-7	Mono-(2-ethylhexyl) phthalate	MEHP	4376-20-9	0.10
		Mono-(2-ethyl-5-hydroxylhexyl) phthalate	oh-MEHP	40321-99-1	0.02
		Mono-(2-ethyl-5-oxohexyl) phthalate	oxo-MEHP	40321-98-0	0.03
		Mono-(2-ethyl-5-carboxypentyl) phthalate	cx-MEPP	40809-41-4	0.02
Diisononyl phthalate (DiNP)	68515-48-0	Mono-(4-methyl-7-hydroxyloctyl) phthalate	oh-MMeOP	936021-98-6	0.02
		Mono-(4-methyl-7-oxo octyl) phthalate	oxo-MMeOP	936022-00-3	0.01
		Mono-(4-methyl-7-carboxyheptyl) phthalate	cx-MMeHP	936022-02-5	0.02

**Figure 1:**
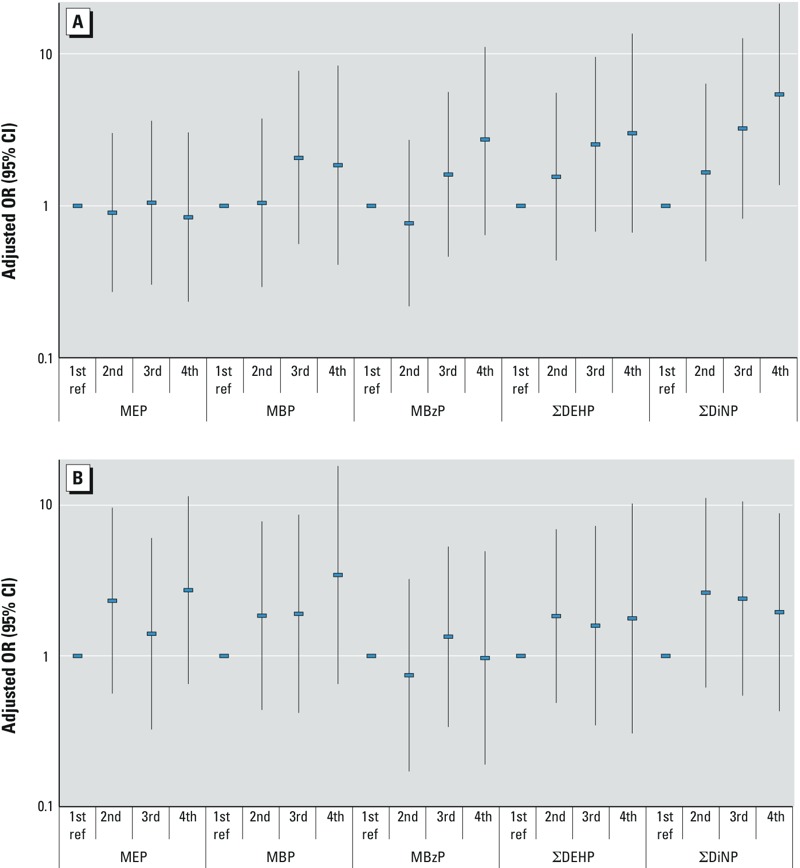
Adjusted odds ratio (OR) for having a short AGD (in the 1st quartile) compared with a long AGD (4th quartile) as a function of quartile of phthalate metabolite concentration in the pregnant women’s urine: (*A*) AGDas; (*B*) AGDap. ref, reference (1st quartile). Adjusted for age (months), gestational week of urine sampling, weight-for-age percentile, and creatinine.

*Statistical analyses*. Urinary levels of phthalate metabolites were log-transformed to normalize distributions and geometric means (GMs) with 95% confidence interval (CIs). Associations between log-transformed phthalate metabolite concentrations and AGD were estimated using a general linear model, and we present the beta coefficients with 95% CI. We also categorized covariate-adjusted AGD (AGDas and AGDap) into “short” (< 25th percentile), “medium” (25th–75th percentile), and “long” (≥ 75th percentile) as a reference. We then used logistic regression models to estimate the odds ratio (OR) of having a boy with a short (adjusted) AGD compared with a long AGD as a function of log-transformed concentrations of phthalate metabolites in prenatal urine. Finally we examined the ORs for having a short AGD compared with a long AGD as a function of quartiles of phthalate metabolite concentration in the pregnant women’s urine, with the lowest quartile as a reference. Therefore, using these models we examined the relation between prenatal phthalate exposure and AGD in three ways: *a*) both AGD and phthalate metabolite concentrations modeled as continuous variables; *b*) categorical AGDs vs. continuous phthalate metabolite concentrations; and *c*) both AGD and phthalate metabolite concentrations modeled as categorical variables (Figure 1).

The literature indicates that both age and body size can influence measures of AGD. Thus, we controlled for body size by using weight-for-age percentiles by the use of WHO standards (WHO 2009). Weight-for-age percentiles are correlated with age, but much less correlated than weight and age (Swan 2008). Finally, in addition to weight-for-age, all models were adjusted for the boy’s age at examination (months), gestational week of urine sampling, and urinary creatinine concentration. A *p*-value < 0.05 was considered statistically significant.

*Ethical approval*. The research ethics committee at Uppsala University, Sweden, approved the study, and informed consent was obtained from all participating adults.

## Results

*Study population.* The study population for this analysis was quite similar to the entire SELMA study population. The 196 families included in the final analysis were somewhat better educated, smoked less, and were more likely to live in a single-family house and in a rural setting (Table 2).

**Table 2:** Characteristics of the study population of 196 boys and the families in the entire SELMA study (%).

Characteristic	Present study (*n *= 196)	SELMA^*a*^ (*n *= 1,974)
Surrounding for the home
Urban	16.8	19.3
Suburban	47.1	47.9
Rural	36.1	32.9
Type of home
Single-family house	64.2	60.8
Row house	7.1	7.9
Apartment in multifamily house	26.4	29.5
Other	2.4	1.8
Education for the mother (highest)
Elementary school	2.8	3.9
High school (Gymnasium)	33.5	36.0
University	58.5	54.0
Other	5.2	6.0
Smoking in family
Any smoker in the family	16.0	18.4
^***a***^Data from a questionnaire at week 10 of pregnancy when urine samples were collected from the pregnant women (*n *= 1,974).		

*AGD.* AGD was measured on 225 boys (19–21 months of age) between June 2011 and August 2012, and prenatal phthalates metabolites were available for 199 of them. Of these, one child was excluded due to an error in weight measurements and two children were excluded due to a CV for the AGD measurements > 10%, resulting in a study population of 196 children. These 196 boys had a mean age of 20.8 months and a mean weight of 12.6 kg. In addition, AGDap was not measured on four of these boys, so for those analyses *n* = 192 (Table 3).The median AGDas was 40.7 mm [interquartile range (IQR) = 37.8–45.0 mm] while the median AGDap was 82.6 mm (IQR = 78.0–87.4 mm). The mean CV was 2.3% for AGDas and 1.6% for AGDap (Table 3).

**Table 3:** Measurements of the 196 boys including AGD and other characteristics.

Characteristic	Mean ± SD	Percentile		
		25th	50th	75th
Age of child (months)	20.8 ± 1.6	19.7	20.7	21.8
Weight of child (kg)	12.6 ± 1.3	11.7	12.6	13.3
Gestational week for urine sampling	10.0 ± 2.3	9.0	10.0	11.0
AGD (mm)
AGDas	41.4 ± 5.98	37.8	40.7	45.0
AGDap (*n *= 192)	82.2 ± 6.94	78.0	82.6	87.4
CV (%)^*a*^
AGDas (*n *= 184)	2.28	1.03	1.59	2.91
AGDap (*n *= 168)	1.58	0.66	1.13	1.84
^***a***^For 12 boys only one measurement was made for AGDas, and for 24 boys only one AGDap measurement was made.				

*Phthalate metabolites.* Ten phthalate metabolites and creatinine were measured in urine collected during weeks 9–11. The GM and 95% CI for the concentration of the 10 metabolites as well as the sum of DEHP and DiNP metabolites are presented in Table 4. All phthalate metabolites were identified above LOD in all urine samples (Table 1). The distribution of the metabolite concentrations in the AGD study population was comparable with the distribution in the entire SELMA cohort (*n* = 2,356) (data not shown). Table 4 also provides unpublished data reflecting urinary levels of phthalate metabolites in pregnant women of boys and girls from the Study for Future Families (SFF), whereas published data reflect boys only (Swan 2008; Swan et al. 2005), as well as from the U.S. National Health and Nutrition Examination Survey (NHANES) study where data are from women of childbearing age [Centers for Disease Control and Prevention (CDC) 2014].

**Table:** Distribution of urinary levels of phthalate metabolites (ng/mL) in 196 women in the present study and in two American studies, SFF and NHANES.

Phthalate	Metabolite	Present study, 2008–2009 (1st trimester) (*n *= 196)				SFF,^*a*^ 2000–2003 (2nd trimester) (*n *= 380)	NHANES,^*b*^ 2005–2010 (women 20–40 years) (*n *= 1,069)
		GM (95% CI)	25th	50th	75th	GM (95% CI)	GM (95% CI)
DEP	MEP	63.64 (54.35, 74.52)	30.67	60.56	134.12	149.00 (124.66, 178.10)	100.99 (89.28, 114.24)
DBP	MBP	67.62 (60.02, 76.17)	43.13	66.03	111.78	15.04 (13.49, 16.77)	18.98 (17.34, 20.77)
BBzP	MBzP	15.99 (13.50, 18.95)	7.85	15.08	35.58	9.97 (8.64, 11.50)	7.37 (6.63, 8.19)
DEHP	MEHP	3.27 (2.87, 3.73)	1.91	3.30	5.86	3.23 (2.82, 3.70)	2.61 (2.31, 2.95)
	oh-MEHP	14.42 (12.82, 16.22)	8.69	15.28	22.85	11.74 (10.28, 13.40)	18.30 (16.14, 20.73)
	oxo-MEHP	9.68 (8.59, 10.91)	5.67	9.99	15.60	10.51 (9.26, 11.93)	11.67 (10.36, 13.14)
	cx-MEPP	14.21 (12.63, 15.98)	8.00	14.53	22.50	19.97 (16.45, 24.24)	28.78 (25.71, 32.21)
	ΣDEHP^*c*^	142.61 (126.99, 160.16)	84.56	148.13	220.71	118.33 (104.37, 134.17)	—
DiNP	oh-MMeOP	6.81 (5.60, 8.28)	2.83	6.27	14.23	—	—
	oxo-MMeOP	3.05 (2.56, 3.63)	1.33	2.75	6.22	—	—
	cx-MMeHP	10.81 (9.16, 12.75)	4.95	8.26	16.43	—	—
	ΣDiNP^*c*^	67.74 (57.00, 80.52)	28.28	55.91	124.92	—	—
Creatinine (mmol/L)	—	9.55 (8.94, 10.20)	7.00	9.60	13.10	—	—
^***a***^Unpublished data from mothers of boys and girls; published data reflect boys only (Swan 2008; Swan et al. 2005). ^***b***^Based on pooled 2005–2010 NHANES cycles, and women 20–40 years of age (CDC 2014). ^***c***^The unit is nmol/L.							

*AGD in relation to phthalate exposure.* Most of the phthalate metabolites were negatively associated with AGD both before and after adjustment for covariates in multiple linear regression models; however, most of the associations did not reach significance. Strongest and most significant inverse associations were found between AGDas and DiNP metabolites and most strongly for oh-MMeOP [mono-(4-methyl-7-hydroxyloctyl) phthalate] and oxo-MMeOP [mono-(2-ethyl-5-oxohexyl) phthalate] and the sum of DiNP metabolites (Table 5).

**Table 5:** Association between AGD in boys and log-transformed concentrations of phthalate metabolites in prenatal urine from an adjusted^*a*^ linear regression model.

Phthalate	Metabolite	AGDas		AGDap	
		β (95% CI)	*p*-Value	β (95% CI)	*p*-Value
DBP	MBP	–1.41 (–4.39, 1.57)	0.351	–2.06 (–5.29, 1.18)	0.211
DEP	MEP	0.63 (–1.29, 2.54)	0.518	–1.30 (–3.40, 0.81)	0.225
BBzP	MBzP	–1.66 (–3.56, 0.25)	0.088	–0.65 (–2.74, 1.44)	0.542
DEHP	MEHP	–1.28 (–3.74, 1.17)	0.304	–1.74 (–4.43, 0.95)	0.203
	oh-MEHP	–1.24 (–3.99, 1.51)	0.374	–1.50 (–4.50, 1.49)	0.324
	oxo-MEHP	–0.77 (–3.48, 1.94)	0.576	–1.25 (–4.19, 1.70)	0.406
	cx-MEPP	–0.89 (–3.69, 1.92)	0.534	–0.64 (–3.69, 2.40)	0.677
	ΣDEHP	–1.16 (–4.01, 1.68)	0.420	–1.39 (–4.49, 1.70)	0.375
DiNP	oh-MMeOP	–1.61 (–3.06, –0.16)	0.029	–1.23 (–2.83, 0.37)	0.131
	oxo-MMeOP	–1.82 (–3.47, –0.17)	0.031	–1.67 (–3.49, 0.15)	0.072
	cx-MMeHP	–1.51 (–3.26, 0.24)	0.091	–1.39 (–3.32, 0.53)	0.156
	ΣDiNP	–1.69 (–3.35, –0.02)	0.047	–1.46 (–3.29, 0.38)	0.119
^***a***^Adjusted for age (months), gestational week of urine sampling, weight-for-age percentile, and creatinine.					

These associations were also seen when AGD was stratified and phthalate concentrations were compared by adjusted AGD quartile (Table 6). The ORs for having an AGDas in the 2nd–3rd quartile when compared with the 4th quartile as a function of (log-transformed) DiNP metabolite concentrations were in the range of 1.1–1.2 but nonsignificant, whereas the ORs for having an AGDas in the 1st quartile when compared with the 4th quartile was in the range of 2.6–3.1 and significant. We also saw weaker inverse associations between AGDap and DINP metabolites, which did not reach statistical significance. Associations between prenatal DEHP metabolite exposure and shorter AGDas were also seen, though these associations did not reach statistical significance.

**Table 6:** Association between AGD (short, medium, and long) and log-transformed concentrations of phthalate metabolites in prenatal urine calculated in an adjusted^*a*^ logistic regression model [OR (95% CI)].

Phthalate	Metabolite	AGDas			AGDap		
		Short 1st quartile	Medium 2nd–3rd quartile	Long 4th quartile (ref)	Short 1st quartile	Medium 2nd–3rd quartile	Long 4th quartile (ref)
DBP	MBP	1.84 (0.43, 7.91)	0.73 (0.21, 2.60)	1.0	1.83 (0.37, 9.02)	0.84 (0.23, 3.01)	1.0
DEP	MEP	1.06 (0.47, 2.40)	0.84 (0.32, 2.16)	1.0	2.23 (0.80, 6.24)	1.11 (0.47, 2.62)	1.0
BBzP	MBzP	1.92 (0.74, 5.00)	0.75 (0.33, 1.79)	1.0	1.49 (0.53, 4.17)	1.21 (0.52, 2.81)	1.0
DEHP	MEHP	1.71 (0.52, 5.64)	1.04 (0.37, 2.93)	1.0	1.98 (0.51, 7.66)	4.01 (1.32, 12.20)	1.0
	oh-MEHP	1.85 (0.50, 6.95)	0.89 (0.27, 2.92)	1.0	2.62 (0.58, 11.78)	3.05 (0.91, 10.16)	1.0
	oxo-MEHP	1.47 (0.40, 5.37)	0.72 (0.22, 2.32)	1.0	2.23 (0.50, 9.87)	3.05 (0.93, 10.07)	1.0
	cx-MEPP	1.76 (0.46, 6.70)	0.98 (0.30, 3.28)	1.0	1.76 (0.38, 8.27)	2.80 (0.82, 9.57)	1.0
	ΣDEHP	1.82 (0.47, 7.06)	0.88 (0.26, 3.00)	1.0	2.44 (0.51, 11.72)	3.36 (0.96, 11.82)	1.0
DiNP	oh-MMeOP	2.61 (1.24, 5.48)	1.08 (0.55, 2.09)	1.0	1.47 (0.67, 3.22)	1.17 (0.61, 2.24)	1.0
	oxo-MMeOP	2.99 (1.28, 7.00)	1.17 (0.54, 2.53)	1.0	1.73 (0.70, 4.23)	1.28 (0.61, 2.70)	1.0
	cx-MMeHP	3.11 (1.27, 7.66)	1.21 (0.52, 2.78)	1.0	1.77 (0.67, 4.65)	1.41 (0.62, 3.21)	1.0
	ΣDiNP	3.02 (1.28, 7.09)	1.16 (0.52, 2.56)	1.0	1.69 (0.68, 4.20)	1.32 (0.61, 2.84)	1.0
ref, reference. ^***a***^Adjusted for age (months), gestational week for urine sampling, weight for age percentile, and creatinine.							

Finally, the odds of having a short AGDas (in the 1st quartile) when compared with a long AGDas (4th quartile) as a function of quartile of phthalate metabolite concentration showed a linear dose–response relationship for prenatal DiNP metabolite exposure (Figure 1A). Similar but somewhat weaker associations were seen between AGDas and DEHP metabolites, as well as well as MBzP metabolites, whereas no associations were seen with MEP and MBP metabolites. No consistent patterns were seen between AGDap and any of the phthalate metabolites (Figure 1B).

## Discussion

Our finding that prenatal phthalate exposure is associated with shorter male AGD is consistent with several earlier studies. However, Swan (2008) found associations between a shorter male AGD and prenatal exposure, particularly for DEHP metabolites, in mothers recruited in 2000–2003, whereas we saw a stronger association with DiNP metabolites. Mothers of the 196 boys included in the current analysis were pregnant in 2009–2010. Phthalates in commerce changed considerably between 2000 and 2010. Since then, DiNP has largely replaced DEHP in soft PVC applications such as flooring materials, and was measureable in 100% of the current mothers. Additionally, in the present study we observed associations primarily with the shorter AGD measure (AGDas) whereas in the SFF study associations were stronger for AGDap (Swan 2008)—a difference that may be attributable partly to subtle differences in measurement methods between the two studies, but is largely unexplained.

The inverse association between prenatal DiNP exposure and AGDas was significant, as expressed in Table 5, but the AGDas reduction was small in relation to the quite large increase in exposure. For example, more than an IQR increase in DiNP exposure (Table 4) was related to a 4% (1.69 mm) reduction in AGDas in the model (Table 5). About the same effect size has been found for DEHP exposure and AGD reduction in the SFF study (Swan 2008). However, such small relative differences have been associated with male genital birth defects and impaired reproductive function in adult males, as described below.

Our finding of an association between AGD and prenatal exposure to DiNP, and to a lesser extent to DEHP (nonsignificant), is not unexpected. DEHP is a known antiandrogen (Foster 2005; Gray et al. 2000), and DiNP may be as well. Prenatal DiNP reduces testosterone production in Harlan rat pups (Hannas et al. 2011), although it is reported to be a less potent antiandrogen than DEHP. Furthermore, DiNP exposure in rats during gestation and perinatally increased the incidence of reproductive malformations in male offspring and caused alterations in fetal testicular testosterone production (Borch et al. 2004; Gray et al. 2000). In addition, DiNP is an analog of DEHP—the reason for replacing DEHP with DiNP in soft PVC is that the two molecules have common chemical properties, which may also include antiandrogenic action. Our finding is also in line with some animal data. Prenatal exposure to DiNP has been found to reduce anogenital distance in male offspring rats in three studies; in Wistar rats (Boberg et al. 2011), in Wistar Imamichi rats (Lee et al. 2006), and in Sprague-Dawley rats (Ostby et al. 2001). In another study by Clewell et al. (2013b), AGD was reported to be reduced, but only on postnatal day 14 and at the highest exposure level. Boberg et al. (2011) found similar dose-related effects of DiNP as those previously shown for DEHP and DBP. They stated that their finding supports the conclusion that DiNP has antiandrogenic properties and is therefore a reproductive toxicant. However, they also stated that DiNP appears to be less potent than DEHP and DBP. On the other hand, in three other studies no association was found between prenatal DiNP exposure and AGD in offspring rats (Clewell et al. 2013a; Gray et al. 2000; Masutomi et al. 2004).

AGD measurements in SELMA boys were longer than those in SFF (Swan 2008; Swan et al. 2005). Median AGDap was 82.6 mm in SELMA and 70.2 mm in SFF; median AGDas was 40.7 mm in SELMA and 36.8 mm in SFF. These differences are at least partly attributable to difference in age at examination; boys in SELMA averaged 20.8 months of age compared with 12.8 months in SFF. The other three studies reporting associations between prenatal phthalate exposure and AGD in offspring children are not comparable with ours because AGD in those studies was measured at birth (Bustamante-Montes et al. 2013; Sathyanarayana et al. 2010; Suzuki et al. 2012).

Prenatal urinary levels of phthalate metabolites in the 196 mothers were comparable with those in the entire SELMA cohort, suggesting that there was no substantial selection bias (data not shown). The urinary levels of most phthalate metabolites are comparable with those in SFF and NHANES, but there are differences. MBP metabolite levels are higher in Swedish pregnant women (SELMA), whereas MEP is higher in pregnant women in the United States (SFF, NHANES). MBzP (monobenzyl phthalate) metabolites levels are slightly higher in Sweden whereas the picture for DEHP metabolites is less clear, with both higher and lower levels in Swedish data (Table 4). There are no data for DiNP metabolites in SFF. However, based on more recent data for the DiNP metabolite cx-MMeHP [mono-(4-methyl-7-carboxyheptyl) phthalate], concentrations in pregnant women in Sweden and the United States were similar in 2008 (Silva et al. 2013).

The associations we observed between prenatal DiNP metabolites and a shorter AGD in baby boys raises concern for at least two reasons. First, anogenital distance has been shown to be associated with adverse health effects in humans. Three studies reported that male infants and boys with hypospadias or undescended testis had reduced AGD (Hsieh et al. 2012; Jain and Singal 2013; Thankamony et al. 2014). Moreover, a shorter AGD in adult men has been related to decreased fertility (Eisenberg et al. 2011), impaired semen quality (Mendiola et al. 2011), and lower serum testosterone levels (Eisenberg et al. 2012). Shortened AGD has also been suggested as a biomarker of testicular dysgenesis syndrome (Sharpe 2005). Second, the use of DiNP is increasing as it is used to replace DEHP in soft PVC, and consequently the human urinary levels of DiNP metabolites are rapidly increasing globally. The total global market for phthalates has been estimated by the ECHA to be about 6 million tons, with 1.4 million tons in Europe, the Middle East, and Africa, 1.1 million tons in the Americas, and 3.5 million tons in Asia, and phthalates represent 84% of the global plasticizer market (ECHA 2013). Of the global market of plasticizers, DiNP and DiDP (diisodecyl phthalate) were reported to represent about 32% (ECHA 2013). Data from the American Chemistry Council (2012) indicate that the annual world production of DiNP was estimated to be about 1.5 million tons in 2013, assuming a production growth of 2.5% during the last years. Biomonitoring data from Europe and the United States also show that human urinary levels of DEHP metabolites are decreasing while DiNP metabolite concentrations are increasing (Göen et al. 2011; Silva et al. 2013).

## Conclusions

The use of DiNP in soft PVC is increasing, and biomonitoring data show that human DiNP metabolite levels are rapidly increasing globally. Our data suggest that this substitute phthalate may not be safer than the chemical it is replacing. We find that DiNP is associated with a shorter AGD in boys at the age of 21 months, which is of concern because AGD has been related to male genital birth defects and impaired reproductive function in adult males.

## References

[r1] Abb M, Heinrich T, Sorkau E, Lorenz W (2009). Phthalates in house dust.. Environ Int.

[r2] American Chemistry Council. (2012). Overview of Our Work and Review of Current Challenges. American Chemistry Council High Phthalates Panel. Presentation at SPI Flexible Vinyl Products 23rd Annual Conference July 2012.. http://www.plasticsindustry.org/files/events/Eileen_ConneelyTuesday.pdf.

[r3] Barret ES Janssen SS Redmon JB Nguyen RHN Kobrosly R Swan SH 2014 Environmental health attitudes and behaviours: findings from a large pregnancy cohort study. Eur J Obstet Gynecol Reprod Biol 176 119 125; 10.1016/j.ejogrb.2014.02.029 24647207 PMC4001243

[r4] Bergh C, Torgrip R, Emenius G, Östman C (2011). Organophosphate and phthalate esters in air and settled dust—a multi-location indoor study.. Indoor Air.

[r5] Bergman Å, Heindel JJ, Jobling S, Kidd KA, Zoeller RT, Jobling SK, eds. (2013). State of the Science of Endocrine Disrupting Chemicals—2012. Geneva:United Nations Environment Programme and World Health Organization.. http://apps.who.int/iris/handle/10665/78101.

[r6] Boberg J, Christiansen S, Axelstad M, Kledal TS, Vinggaard AM, Dalgaard M (2011). Reproductive and behavioral effects of diisononyl phthalate (DINP) in perinatally exposed rats.. Reprod Toxicol.

[r7] Borch J, Ladefoged O, Hass U, Vinggaard AM (2004). Steroidogenesis in fetal male rats is reduced by DEHP and DINP, but endocrine effects of DEHP are not modulated by DEHA in fetal, prepubertal and adult male rats.. Reprod Toxicol.

[r8] Bornehag CG Lundgren B Weschler CJ Sigsgaard T Hagerhed-Engman L Sundell J 2005 Phthalates in indoor dust and their association with building characteristics. Environ Health Perspect 113 1399 1404; 10.1289/ehp.7809 16203254 PMC1281287

[r9] Bornehag CG, Moniruzzaman S, Larsson M, Lindström CB, Hasselgren M, Bodin A (2012). The SELMA study: a birth cohort study in Sweden following more than 2000 mother–child pairs.. Paediatr Perinat Epidemiol.

[r10] Bornehag CG Sundell J Weschler CJ Sigsgaard T Lundgren B Hasselgren M 2004 The association between asthma and allergic symptoms in children and phthalates in house dust: a nested case–control study. Environ Health Perspect 112 1393 1397; 10.1289/ehp.7187 15471731 PMC1247566

[r11] Bustamante-Montes L Hernández-Valero M Flores-Pimentel D Garcıa-Fábila M Amaya-Chávez A Barr D 2013 Prenatal exposure to phthalates is associated with decreased anogenital distance and penile size in male newborns. J Dev Orig Health Dis 4 4 300 306; 10.1017/S2040174413000172 24349678 PMC3862078

[r12] Carlstedt F, Jönsson B, Bornehag CG (2013). PVC flooring is related to human uptake of phthalates in infants.. Indoor Air.

[r13] CDC (Centers for Disease Control and Prevention). (2014). National Health and Nutrition Examination Survey: NHANES 2009–2010.. http://wwwn.cdc.gov/nchs/nhanes/search/nhanes09_10.aspx.

[r14] Clewell RA, Sochaski M, Edwards K, Creasy DM, Willson G, Andersen ME (2013a). Disposition of diiosononyl phthalate and its effects on sexual development of the male fetus following repeated dosing in pregnant rats.. Reprod Toxicol.

[r15] Clewell RA, Thomas A, Willson G, Creasy DM, Andersen ME (2013b). A dose response study to assess effects after dietary administration of diisononyl phthalate (DINP) in gestation and lactation on male rat sexual development.. Reprod Toxicol.

[r16] Dodson R, Nishioka M, Standley L, Perovich L, Brody J, Rudel R (2011). Chemical analysis of household and personal care products for endocrine disrupting compounds and other chemicals of emerging concern [Abstract]. Epidemiology.

[r17] ECHA (European Chemicals Agency). (2013). Evaluation of New Scientific Evidence Concerning DINP and DIDP in Relation to Entry 52 of Annex XVII to Reach Regulation (EC) No 1907/2006 (Final Review Report).. http://echa.europa.eu/documents/10162/31b4067e-de40-4044-93e8-9c9ff1960715.

[r18] Eisenberg ML Hsieh MH Walters RC Krasnow R Lipshultz LI 2011 The relationship between anogenital distance, fatherhood, and fertility in adult men. PLoS One 6 5 e18973; 10.1371/journal.pone.0018973 21589916 PMC3092750

[r19] Eisenberg ML, Jensen TK, Walters RC, Skakkebaek NE, Lipshultz LI (2012). The relationship between anogenital distance and reproductive hormone levels in adult men.. J Urol.

[r20] Foster PMD (2005). Disruption of reproductive development in male rat offspring following in utero exposure to phthalate esters.. Int J Androl.

[r21] Frederiksen H, Jørgensen N, Andersson A (2010). Correlations between phthalate metabolites in urine, serum, and seminal plasma from young Danish men determined by isotope dilution liquid chromatography tandem mass spectrometry.. J Anal Toxicol.

[r22] Fromme H, Gruber L, Seckin E, Raab U, Zimmermann S, Kiranoglu M (2011). Phthalates and their metabolites in breast milk—results from the Bavarian Monitoring of Breast Milk (BAMBI).. Environ Int.

[r23] Göen T, Dobler L, Koschorreck J, Müller J, Wiesmüller GA, Drexler H (2011). Trends of the internal phthalate exposure of young adults in Germany—follow-up of a retrospective human biomonitoring study.. Int J Hyg Environ Health.

[r24] Gray LE, Ostby J, Furr J, Price M, Veeramachaneni DR, Parks L (2000). Perinatal exposure to the phthalates DEHP, BBP, and DINP, but not DEP, DMP, or DOTP, alters sexual differentiation of the male rat.. Toxicol Sci.

[r25] Hannas BR, Lambright CS, Furr J, Howdeshell KL, Wilson VS, Gray LE (2011). Dose-response assessment of fetal testosterone production and gene expression levels in rat testes following *in utero* exposure to diethylhexyl phthalate, diisobutyl phthalate, diisoheptyl phthalate, and diisononyl phthalate.. Toxicol Sci.

[r26] Hsieh MH, Eisenberg ML, Hittelman AB, Wilson JM, Tasian GE, Baskin LS (2012). Caucasian male infants and boys with hypospadias exhibit reduced anogenital distance.. Hum Reprod.

[r27] Huang PC, Kuo PL, Chou YY, Lin SJ, Lee CC (2009). Association between prenatal exposure to phthalates and the health of newborns.. Environ Int.

[r28] Jain VG, Singal AK (2013). Shorter anogenital distance correlates with undescended testis: a detailed genital anthropometric analysis in human newborns.. Hum Reprod.

[r29] Jensen MS Nørgaard-Pedersen B Toft G Hougaard DM Bonde JP Cohen A 2012 Phthalates and perfluorooctanesulfonic acid in human amniotic fluid: temporal trends and timing of amniocentesis in pregnancy. Environ Health Perspect 120 897 903; 10.1289/ehp.1104522 22398305 PMC3385442

[r30] Koch HM, Lorber M, Christensen KL, Pälmke C, Koslitz S, Brüning T (2013). Identifying sources of phthalate exposure with human biomonitoring: results of a 48h fasting study with urine collection and personal activity patterns.. Int J Hyg Environ Health.

[r31] Langer S, Bekö G, Weschler CJ, Brive LM, Toftum J, Callesen M (2014). Phthalate metabolites in urine samples from Danish children and correlations with phthalates in dust samples from their homes and daycare centers.. Int J Hyg Environ Health.

[r32] Langer S, Weschler CJ, Fischer A, Bekö G, Toftum J, Clausen G (2010). Phthalate and PAH concentrations in dust collected from Danish homes and daycare centers.. Atmos Environ.

[r33] Lee H, Yamanouchi K, Nishihara M (2006). Effects of perinatal exposure to phthalate/adipate esters on hypothalamic gene expression and sexual behavior in rats.. J Reprod Dev.

[r34] MacLeod DJ, Sharpe RM, Welsh M, Fisken M, Scott HM, Hutchison GR (2010). Androgen action in the masculinization programming window and development of male reproductive organs.. Int J Androl.

[r35] Masutomi N, Shibutani M, Takagi H, Uneyama C, Lee K, Hirose M (2004). Alteration of pituitary hormone-immunoreactive cell populations in rat offspring after maternal dietary exposure to endocrine-active chemicals.. Arch Toxicol.

[r36] Mazzachi BC, Peake MJ, Ehrhardt V (2000). Reference range and method comparison studies for enzymatic and Jaffé creatinine assays in plasma and serum and early morning urine.. Clin Lab.

[r37] Mendiola J Stahlhut RW Jørgensen N Liu F Swan SH 2011 Shorter anogenital distance predicts poorer semen quality in young men in Rochester, New York. Environ Health Perspect 119 958 963; 10.1289/ehp.1103421 21377950 PMC3222997

[r38] Ostby J, Hotchkiss A, Furr J, Gray L (2001). Investigation of the ability of diisononyl phthalate (DINP) to alter androgen-dependent tissue development in Sprague-Dawley rats [Abstract]. Toxicologist.

[r39] Rudel RA Gray JM Engel CL Rawsthorne TW Dodson RE Ackerman JM 2011 Food packaging and bisphenol A and bis (2-ethyhexyl) phthalate exposure: findings from a dietary intervention. Environ Health Perspect 119 914 920; 10.1289/ehp.1003170 21450549 PMC3223004

[r40] Rudel RA, Perovich LJ (2009). Endocrine disrupting chemicals in indoor and outdoor air.. Atmos Environ.

[r41] Sathyanarayana S, Beard L, Zhou C, Grady R (2010). Measurement and correlates of ano-genital distance in healthy, newborn infants.. Int J Androl.

[r42] Sharpe RM (2005). Phthalate exposure during pregnancy and lower anogenital index in boys: wider implications for the general population? [Editorial]. Environ Health Perspect.

[r43] Sharpe RM, Fisher JS, Millar MM, Jobling S, Sumpter JP (1995). Gestational and lactational exposure of rats to xenoestrogens results in reduced testicular size and sperm production.. Environ Health Perspect.

[r44] Shi W, Hu X, Zhang F, Hu G, Hao Y, Zhang X (2012). Occurrence of thyroid hormone activities in drinking water from eastern China: contributions of phthalate esters.. Environ Sci Technol.

[r45] Silva MJ, Jia T, Samandar E, Preau JL, Calafat AM (2013). Environmental exposure to the plasticizer 1,2-cyclohexane dicarboxylic acid, diisononyl ester (DINCH) in U.S. adults (2000–2012).. Environ Res.

[r46] Suzuki Y, Yoshinaga J, Mizumoto Y, Serizawa S, Shiraishi H (2012). Foetal exposure to phthalate esters and anogenital distance in male newborns.. Int J Androl.

[r47] Swan SH (2008). Environmental phthalate exposure in relation to reproductive outcomes and other health endpoints in humans.. Environ Res.

[r48] Swan SH Main KM Liu F Stewart SL Kruse RL Calafat AM 2005 Decrease in anogenital distance among male infants with prenatal phthalate exposure. Environ Health Perspect 113 1056 1061; 10.1289/ehp.8100 16079079 PMC1280349

[r49] Thankamony A Lek N Carroll D Williams M Dunger DB Acerini CL 2014 Anogenital distance and penile length in infants with hypospadias or cryptorchidism: comparison with normative data. Environ Health Perspect 122 207 211; 10.1289/ehp.1307178 24316680 PMC3915266

[r50] U.S. Environmental Protection Agency. (1996). Guidelines for Reproductive Toxicity Risk Assessment. Fed Reg 61(212):56274–56322.. http://www.epa.gov/raf/publications/pdfs/REPRO51.PDF.

[r51] van den Driesche S Scott HM MacLeod DJ Fisken M Walker M Sharpe RM 2011 Relative importance of prenatal and postnatal androgen action in determining growth of the penis and anogenital distance in the rat before, during and after puberty. Int J Andrology, 34 6 Pt 2 e578 e586 10.1111/j.1365-2605.2011.01175.x 21631528

[r52] Wan HT, Leung PY, Zhao YG, Wei X, Wong MH, Wong CK (2013). Blood plasma concentrations of endocrine disrupting chemicals in Hong Kong populations.. J Hazard Mater.

[r53] WHO (World Health Organization). (2009). WHO Child Growth Standards: Growth Velocity Based on Weight, Length and Head Circumference: Methods and Development. Geneva:WHO, Department of Nutrition for Health and Development.. http://apps.who.int/iris/bitstream/10665/44026/1/9789241547635_eng.pdf?ua.

[r54] Wittassek M, Koch HM, Angerer J, Brüning T (2011). Assessing exposure to phthalates—the human biomonitoring approach.. Mol Nutr Food Res.

[r55] Zhang Q, Lu XM, Zhang XL, Sun YG, Zhu DM, Wang BL (2013). Levels of phthalate esters in settled house dust from urban dwellings with young children in Nanjing, China.. Atmos Environ.

